# Supplementary material for: In Silico Molecular Analysis of Carbapenemase-Negative Carbapenem-Resistant Pseudomonas aeruginosa Strains in Greece
Source: Microorganisms. 2024 Apr 16;12(4):805. doi: 10.3390/microorganisms12040805 (PMC11051870; doi:10.3390/microorganisms12040805)
Supplement: Supplementary file 1 [file microorganisms-12-00805-s001.zip › microorganisms-2939063-supplementary.pdf]

In Silico Molecular Analysis of Carbapenemase-Negative Carbapenem-Resistant *Pseudomonas aeruginosa* Strains in Greece

Katerina Tsilipounidaki, Christos-Georgios Gkountinoudis, Zoi Florou, George C. Fthenakis, and Efthymia Petinaki

Figure S1: Diagram of the transcriptional regulatory mechanisms controlling MexAB-OprM efflux pumps in *Pseudomonas aeruginosa*: transcriptional repression of the mexAB-oprM operon is mediated directly by MexR and NalD proteins and indirectly by NalC, which represses ArmR protein, an anti-repressor of MexR.

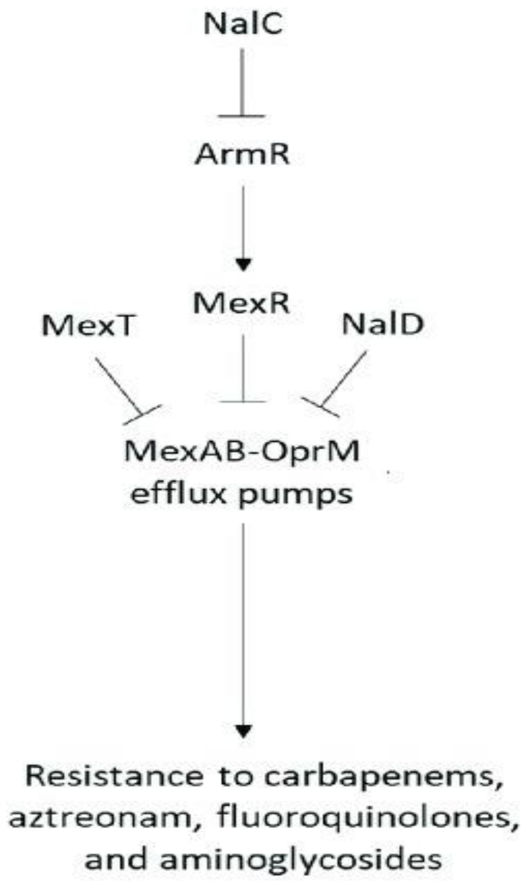

Table S1: Sequences of the *Pseudomonas aeruginosa* strains, aligned with ClustalW (using default parameters), where occurrences of frame shifts or stop codons have been detected. Premature stop codons are visually highlighted in red, while the first nucleotide or gap signifying a frame shift is highlighted in green. In *oprD* gene, premature stops codons were detected in strains 3, 6, 20 and 21, while in strain 8, a deletion of 20 amino acids was detected due to the presence of a premature stop codon. In the *nalC* gene, strain 18 exhibited a premature stop codon, whereas strain 19 displayed a frame shift was detected at position 18. In the *mexR* gene, a premature stop codon was detected in strain 1; in strain 18 exhibited a frame shift, due to a 7-nucleotide gap at position 266.

oprD

```
CLUSTAL 2.1 multiple sequence alignment


PAO1            ATGAAAGTGATGAAGTGGAGCGCCATTGCACTGGCGGTTTCCGCAGGTAGCACTCAGTTC
8               ATGAAAGTGATGAAGTGGAGCGCCATTGCACTGGCGGTTTCCGCAGGTAGCACTCAGTTC
20              ATGAAAGTGATGAAGTGGAGCGCCATTGCACTGGCGGTTTCCGCAGGTAGCACTCAGTTC
6               ATGAAAGTGATGAAGTGGAGCGCCATTGCACTGGCGGTTTCCGCAGGTAGCACTCAGTTC
3               ATGAAAGTGATGAAGTGGAGCGCCATTGCACTGGCGGTTTCCGCAGGTAGCACTCAGTTC
21              ATGAAAGTGATGAAGTGGAGCGCCATTGCACTGGCGGTTTCCGCAGGTAGCACTCAGTTC
                ************************************************************

PAO1            GCCGTGGCCGACGCATTCGTCAGCGATCAGGCCGAAGCGAAGGGGTTCATCGAAGACAGC
8               GCCGTGGCCGACGCATTCGTCAGCGATCAGGCCGAAGCGAAGGGGTTCATCGAAGACAGC
20              GCCGTGGCCGACGCATTCGTCAGCGATCAGGCCGAAGCGAAGGGGTTCATCGAAGACAGC
6               GCCGTGGCCGACGCATTCGTCAGCGATCAGGCCGAAGCGAAGGGGTTCATCGAAGACAGC
3               GCCGTGGCCGACGCATTCGTCAGCGATCAGGCCGAAGCGAAGGGGTTCATCGAAGACAGC
21              GCCGTGGCCGACGCATTCGTCAGCGATCAGGCCGAAGCGAAGGGGTTCATCGAAGACAGC
                ************************************************************

PAO1            AGCCTCGACCTGCTGCTCCGCAACTACTATTTCAACCGTGACGGCAAGAGCGGCAGCGGG
8               AGCCTCGACCTGCTGCTCCGCAACTACTATTTCAACCGTGACGGCAAGAGCGGCAGCGGG
20              AGCCTCGACCTGCTGCTCCGCAACTACTATTTCAACCGTGACGGCAAGAGCGGCAGCGGG
6               AGCCTCGACCTGCTGCTCCGCAACTACTATTTCAACCGTGACGGCAAGAGCGGCAGCGGG
3               AGCCTCGACCTGCTGCTCCGCAACTACTATTTCAACCGTGACGGCAAGAGCGGCAGCGGG
21              AGCCTCGACCTGCTGCTCCGCAACTACTATTTCAACCGTGACGGCAAGAGCGGCA-CGGG
                ******************************************************* ****

PAO1            GACCGCGTCGACTGGACCCAAGGCTTCCTCACCACCTATGAATCCGGCTTCACCCAAGGC
8               GACCGCGTCGACTGGACCCAAGGCTTCCTCACCACCTATGAATCCGGCTTCACTCAAGGC
20              GACCGCGTCGACTGGACC-AAGGCTTCCTCACCACCTATGAATCCGGCTTCACCCAAGGC
6               GACCGCGTCGACTGGACCCAAGGCTTCCTCACCACCTATGAATCCGGCTTCACCCAAGGC
3               GACCGCGTCGACTGGACCCAAGGCTTCCTCACCACCTATGAATCCGGCTTCACCCAAGGC
21              GACCGCGTCGACTGGACCCAAGGCTTCCTCACCACCTATGAATCCGGCTTCACCCAAGGC
                ****************** ********************************** ******
```

```
PAO1          ACTGTGGGCTTCGGCGTCGATGCCTTCGGCTACCTGGGCCTGAAGCTCGACGGCACCTCC
8             ACCGTGGGCTTCGGCGTCGATGCCTTCGGCTACCTGGGCCTGAAGCTCGACGGCACCTCC
20            ACCGTGGGCTTCGGCGTCGATGCCTTCGGCTACCTCGGCCTGAAGCTCGACGGCACCTCG
6             ACTGTGGGCTTCGGCGTCGATGCCTTCGGCTACCTCGGCCTGAAGCTCGACGGTACCTCT
3             ACCGTGGGCTTCGGCGTCGATGCCTTCGGCTACCTCGGTCTGAAGCTCGACGGCACCTCG
21            ACCGTGGGCTTCGGCGTCGATGCCTTCGGCTACCTCGGTCTGAAGCTCGACGGCACCTCG
              ** ******************************** ** ************** *****

PAO1          GACAAGACCGGCACCGGCAACCTGCCGGTGATGAACGACGGCAAGCCGCGCGATGACTAC
8             GACAAGACCGGCACCGGCAACCTGCCGGTGATGAACGACGGCAAGCCGCGCGATGACTAC
20            GACAAGACCGGCACCGGCAACCTGCCGGTGATGAACGACGGCAAGCCGCGCGACGACTAC
6             GACAAGAGCGGCACCGGCAACCTGCCAGTAATGAACGACGGAACGCCCCGTGACGACTAC
3             GACAAGAGCGGTACCGGCAACCTGCCGGTGATGAACGACGGCACGCCCCGTGACGACTAC
21            GACAAGAGCGGTACCGGCAACCTGCCGGTGATGAACGACGGCACGCCCCGTGACGACTAC
              ******* *** ************** ** *********** * *** ** ** ******

PAO1          AGCCGCGCCGGCGGCGCCGTGAAGGTGCGCATCTCCAAGACCATGCTGAAGTGGGGCGAG
8             AGCCGCGCCGGCGGCGCCGTGAAGGTGCGCATCTCCAAGACCATGCTGAAGTGGGGCGAG
20            AGCCGCGCTGGCGGCGCCCTGAAGGTGCGCATTTCCAAGACCATGCTGAAGTGGGGCGAA
6             AGCCGCGCCGGTGGCGCCGTGAAGGTACGCATCTCCAAGACCATGCTGAAGTGGGGCGAG
3             AGCCGCGCCGGTGGCGCCGTGAAGGTACGCATCTCCAAGACCATGTTGAAGTGGGGCGAG
21            AGCCGCGCCGGTGGCGCCGTGAAGGTACGCATCTCCAAGACCATGTTGAAGTGGGGCGAG
              ******** ** ****** ******* ***** ************ *************

PAO1          ATGCAACCGACCGCCCCGGTCTTCGCCGCTGGCGG-CAGCCGCCTGTTCCCGCAGACCGC
8             ATGCAACCGACCGCCCCGGTCTTCGCCGCTGGCGG-CAGCCGCCTGTTCCCGCAGACCGC
20            ATGCAACCTACCGCGCCGGTCTTCGCCGCCGGCGG-CAGCCGCCTGTTCCCGCAGACCGC
6             ATGCAGCCGACCGCTCCGGTCTTCGCCGCTGGGGGGCAGCCGCCTGTTCCCCCAGACCGC
3             ATGTAGCCGACCGCTCCGGTCTTCGCCGCCGGCGG-CAGCCGCCTGTTCCCGCAGACCGC
21            ATGCAGCCGACCGCTCCGGTCTTCGCCGCCGGCGG-CAGCCGCCTGTTCCCGCAGACCGC
              *** * ** ***** ************** ** ** *************** ********

PAO1          GACCGGCTTCCAGCTGCAGAGCAGCGAATTCGAAGGGCTCGACCTCGAGGCAGGCCACTT
8             GACCGGCTTCCAGCTGCAGAGCAGCGAATTCGAAGGGCTCGACCTCGAGGCAGGCCACTT
20            GACCGGCTTCCAACTGCAGAGCAGTGAATTCGAAGGGCTCGATCTCGAAGCGGGCCACTT
6             GACCGGCTTCCAGCTGCAGAGCAGCGAACTCGAAGGGCTCGACCTCGAGGCAGGCCACTT
3             GACCGGCTTCCAACTGCAGAGCAGCGAACTCGAAGGGCTCGATCTCGAAGCGGGCCACTT
21            GACCGGCTTCCAACTGCAGAGCAGCGAACTCGAAGGGCTCGATCTCGAAGCGGGCCACTT
              ************ *********** *** ************* ***** ** ********
```

```
PAO1    CACCGAGGGCAAGGAGCCGACCACCGTCAAATCGCGTGGCGAACTCTATGCCACCTACGC
8       CACCGAGGGCAAGGAGCCGACCACCGTCAAATCGCGTGGCGAACTCTATGCCACCTACGC
20      CACCGAGGGCAAGGAGCCGACCACCGTCAAATCGCGTGGCGAACTCTATGCCACCTACGC
6       CACCGAGGGCAAGGAGCCGACCACCGTCAAATCGCGTGGCGAACTCTATGCCACCTACGC
3       CACCGAAGGCAAGCAGGGCACCACCACCAAGTCGCGCGGCGAACTCTACGCAACCTATGC
21      CACCGAAGGCAAGCAGGGCACCACCACCAAGTCGCGCGGCGAACTCTACGCAACCTATGC
        ****** ****** **   ******  *** ***** *********** ** ***** **

PAO1    AGGCGAGACCGCCAAGAGCGCCGATTTCATTGGGGGCCGCTACGCAATCACCGATAACCT
8       AGGCGAGACCGCCAAGAGCGCCGATTTCATTGGGGGCCGCTACGCAATCACCGATAACCT
20      AGGCGAGACCGCCAAGAGCGCCGATTTCATTGGGGGCCGCTACGCAATCACCGATAACCT
6       AGGCGAGACCGCCAAGAGCGCCGATTTCATTGGGGGCCGCTACGCAATCACCGATAACCT
3       AGGCGAGACCGCCAAGAGCGCCGATTTCATTGGGGGCCGCTACGCAATCACCGATAACCT
21      AGGCGAGACCGCCAAGAGCGCCGATTTCATTGGGGGCCGCTACGCAATCACCGATAACCT
        ************************************************************

PAO1    CAGCGCCTCCCTGTACGGCGCCGAACTCGAAGACATCTATCGCCAGTATTACCTGAACAG
8       CAGCGCCTCCCTGTACGGCGCCGAACTCGAAGACATCTATCGCCAGTATTACCTGAACAG
20      CAGCGCCTCCCTGTACGGCGCCGAACTCGAAGACATCTATCGCCAGTATTACCTGAACAG
6       CAGCGCCTCCCTGTACGGCGCCGAACTCGAAGACATCTATCGCCAGTATTACCTGAACAG
3       CAGCGCCTCCCTGTACGGTGCTGAACTCGAAGACATCTATCGTCAGTATTACCTGAACAG
21      CAGCGCCTCCCTGTACGGTGCTGAACTCGAAGACATCTATCGTCAGTATTACCTGAACAG
        ****************** ** ******************** *****************

PAO1    CAACTACACCATCCCACTGGCATCCGACCAATCGCTGGGCTTCGATTTCAACATCTACCG
8       CAACTACACCATCCCACTGGCATCCGACCAATCGCTGGGCTTCGATTTCAACATCTACCG
20      CAACTACACCATCCCACTGGCATCCGACCAATCGCTGGGCTTCGATTTCAACATCTACCG
6       CAACTACACCATCCCACTGGCATCCGACCAATCGCTGGGCTTCGATTTCAACATCTACCG
3       CAACTACACCATCCCACTGGCATCCGACCAATCGCTGGGCTTCGATTTCAACATCTACCG
21      CAACTACACCATCCCACTGGCATCCGACCAATCGCTGGGCTTCGATTTCAACATCTACCG
        ************************************************************

PAO1    CACAAACGATGAAGGCAAGGCCAAGGCCGGCGACATCAGCAACACCACTTGGTCCCTGGC
8       CACAAACGATGAAGGCAAGGCCAAGGCCGGCGACATCAGCAACACCACTTGGTCCCTGGC
20      CACAAACGATGAAGGCAAGGCCAAGGCCGGCGACATCAGCAACACCACTTGGTCCCTGGC
6       CACAAACGATGAAGGCAAGGCCAAGGCCGGCGACATCAGCAACACCACTTGGTCCCTGGC
3       CACAAACGATGAAGGCAAGGCCAAGGCCGGCGACATCAGCAACACCACTTGGTCCCTGGC
21      CACAAACGATGAAGGCAAGGCCAAGGCCGGCGACATCAGCAACACCACTTGGTCCCTGGC
        ************************************************************
```

```
PAO1    GGCAGCCTACACTCTGGATGCGCACACTTTCACCTTGGCCTACCAGAAGGTCCATGGCGA
8       GGCAGCCTACACTCTGGATGCGCACACTTTCACCTTGGCCTACCAGAAGGTCCATGGCGA
20      GGCAGCCTACACTCTGGATGCGCACACTTTCACCTTGGCCTACCAGAAGGTCCATGGCGA
6       GGCAGCCTACACTCTGGATGCGCACACTTTCACCTTGGCCTACCAGAAGGTCCATGGCGA
3       GGCAGCCTACACTCTGGATGCGCACACTTTCACCTTGGCCTACCAGAAGGTCCATGGCGA
21      GGCAGCCTACACTCTGGATGCGCACACTTTCACCTTGGCCTACCAGAAGGTCCATGGCGA
        ************************************************************

PAO1    TCAGCCGTTTGATTATATCGGCTTCGGCCGCAACGGCTCTGGCGCAGGTGGCGACTCGAT
8       TCAGCCGTTTGATTATATCGGCTTCGGCCGCAACGGCTCTGGCGCAGGTGGCGACTCGAT
20      TCAGCCGTTTGATTATATCGGCTTCGGCCGCAACGGCTCTGGCGCAGGTGGCGACTCGAT
6       TCAGCCGTTTGATTATATCGGCTTCGGCCGCAACGGCTCTGGCGCAGGTGGCGACTCGAT
3       TCAGCCGTTTGATTATATCGGCTTCGGCGAGAACGGTTCCGGCGGCGGCGGTGACTCGAT
21      TCAGCCGTTTGATTATATCGGCTTCGGCGAGAACGGTTCCGGCGGCGGCGGTGACTCGAT
        ****************************   ***** ** ****  ** ** ********

PAO1    TTTCCTCGCCAACTCTGTCCAGTACTCCGACTTCAACGGCCCTGGCGAGAAATCCTGGCA
8       TTTCCTCGCCAACTCTGTCCAGTACTCCGACTTCAACGGCCCTGGCGAGAAATCCTGGCA
20      TTTCCTCGCCAACTCTGTCCAGTACTCCGACTTCAACGGCCCTGGCGAGAAATCCTGGCA
6       TTTCCTCGCCAACTCTGTCCAGTACTCCGACTTCAACGGCCCTGGCGAGAAATCCTGGCA
3       TTTCCTCGCCAACTCCGTGCAGTACTCCGACTTCAACGGCCCCGGCGAGAAATCCTGGCA
21      TTTCCTCGCCAACTCCGTGCAGTACTCCGACTTCAACGGCCCCGGCGAGAAATCCTGGCA
        *************** ** *********************** *****************

PAO1    GGCTCGCTACGACCTGAACCTAGCCTCCTATGGCGTTCCCGGCCTGACTTTCATGGTCCG
8       GGCTCGCTACGACCTGAACCTAGCCTCCTATGGCGTTCCCGGCCTGACTTTCATGGTCCG
20      GGCTCGCTACGACCTGAACCTAGCCTCCTATGGCGTTCCCGGCCTGACTTTCATGGTCCG
6       GGCTCGCTACGACCTGAACCTAGCCTCCTATGGCGTTCCCGGCCTGACTTTCATGGTCCG
3       GGCCCGCTACGACCTGAACCTCGCCTCCTATGGCGTTCCCGGCCTGACTTTCATGGTCCG
21      GGCCCGCTACGACCTGAACCTCGCCTCCTATGGCGTTCCCGGCCTGACTTTCATGGTCCG
        *** ***************** **************************************

PAO1    CTATATCAATGGCAAGGACATCGATGGCACCAAGATGTCTGACAACAACGTCGGCTATAA
8       CTATATCAATGGCAAGGACATCGATGGCACCAAGATGTCTGACAACAACGTCGGCTATAA
20      CTATATCAATGGCAAGGACATCGATGGCACCAAGATGTCTGACAACAACGTCGGCTATAA
6       CTATATCAATGGCAAGGACATCGATGGCACCAAGATGTCTGACAACAACGTCGGCTATAA
3       CTATATCAATGGCAAGGACATCGATGGCACCAAGATGTCTGACAACAACGTCGGCTATAA
21      CTATATCAATGGCAAGGACATCGATGGCACCAAGATGTCTGACAACAACGTCGGCTATAA
        ************************************************************
```

```
PAO1            GAACTACGGCTACGGCGAGGATGGCAAGCACCACGAAACCAACCTCGAAGCCAAGTACGT
8               GAACTACGGCTACGGCGAGGATGGCAAGCACCACGAAACCAACCTCGAAGCCAAGTACGT
20              GAACTACGGCTACGGCGAGGACGGCAAGCACCACGAGACCAACCTCGAAGCCAAGTACGT
6               GAACTACGGCTACGGCGAGGACGGCAAGCACCACGAGACCAACCTCGAAGCCAAGTACGT
3               GAACTACGGCTACGGCGAGGACGGCAAGCACCACGAGACCAACCTGGAAGCCAAGTACGT
21              GAACTACGGCTACGGCGAGGACGGCAAGCACCACGAGACCAACCTGGAAGCCAAGTACGT
                ********************* ************** ******** **************

PAO1            GGTCCAGTCCGGTCCGGCCAAGGACCTGTCGTTCCGCATCCGCCAGGCCTGGCACCGTGC
8               GGTCCAGTCCGGTCCGGCCAAGGACCTGTCGTTCCGCATCCGCCAGGCCTGGCACCGTGC
20              GGTCCAGTCCGGTCCGGCCAAGGACCTGTCGTTCCGCATCCGCCAGGCCTGGCACCGTGC
6               GGTCCAGTCCGGTCCGGCCAAGGACCTGTCGTTCCGCATCCGCCAGGCCTGGCACCGTGC
3               GGTCCAGTCCGGTCCGGCCAAGGACCTGTCGTTCCGCATCCGCCAGGCCTGGCACCGCGC
21              GGTCCAGTCCGGTCCGGCCAAGGACCTGTCGTTCCGCATCCGCCAGGCCTGGCACCGCGC
                ********************************************************* **

PAO1            CAACGCCGACCAGGGCGAAGGCGACCAGAACGAGTTCCGCCTGATCGTCGACTATCCGCT
8               CAACGCCGACTAGGGCGAAGGCGACCAGAACGAGTTCCGCCTGATCGTCGACTATCCGCT
20              CAACGCCGACCAGGGCGAAGGCGACCAGAACGAGTTCCGCCTGATCGTCGACTATCCGCT
6               CAACGCCGACCAGGGCGAAGGCGACCAGAACGAGTTCCGCCTGATCGTCGACTATCCGCT
3               CAACGCCGACCAGGCCGAAGGCGACCAGAACGAGTTCCGCCTGATCGTCGACTATCCGCT
21              CAACGCCGACCAGGCCGAAGGCGACCAGAACGAGTTCCGCCTGATCGTCGACTATCCGCT
                ********** *** *********************************************

PAO1            GTCGATCCTGTAA
8               GTCGATCCTGTAA
20              GTCGATCCTGTAA
6               GTCGATCCTGTAA
3               GTCGATCCTGTAA
21              GTCGATCCTGTAA
                *************
```

nalC

```
CLUSTAL 2.1 multiple sequence alignment

PAO1            ATGAACGATGC-TTCTCCCCGTCTGACCGAACGCGGCAGGCAACGCCGCCGCGCCATGCT
19              ATGAACGATGCGTCCCCCCCGTCTGACCGAACGCGGCAGGCAACGCCGCCGCGCCATGCT
18              ATGAACGATGCGTCCCCCCCGTCTGACCGAACGCGGCAGGCAACGCCGCCGCGCCATGCT
                *********** * * ********************************************
```

```
PAO1            CGACGCCGCTACCCAGGCCTTTCTCGAACACGGTTTCGAAGGCACCACCCTGGACATGGT
19              CGACGCCGCTACCCAGGCCTTTCTCGAACACGGTTTCGAAGGCACCACCCTGGACATGGT
18              CGACGCCGCCACCCAGGCCTTTCTCGAACACGGTTTCGAAGGCACCACCCTGGACATGGT
                ********* **************************************************

PAO1            GATAGAACGGGCCGGTGGTTCACGGGGGACCCTGTACAGCTCCTTCGGCGGCAAGGAGGG
19              GATAGAACGGGCCGGTGGTTCACGGGGGACCCTGTACAGCTCCTTCGGCGGCAAGGAGGG
18              GATTGAACGGGCCGGTGGTTCACGGGGAACCCTGTACAGCTCCTTCGGCGGCAAGGAAGG
                *** *********************** ***************************** **

PAO1            CCTGTTCGCCGCGGTGATCGCCCACATGATCGGGGAAATCTTCGACGACAGCGCCGATCA
19              CCTGTTCGCCGCGGTGATCGCCCACATGATCGAGGAAATCTTCGACGACAGCGCCGATCA
18              CCTGTTTGCCGCGGTGATCGCCCACATGATCGAGGAAATCTTCGACGACAGCGCCGATCA
                ****** ************************* ***************************

PAO1            GCCGCGCCCCGCCGCCACGCTGAGCGCCACCCTCGAGCATTTCGGCCGGCGCTTTCTCAC
19              GCCGCGCCCCGCCGCCACGCTGAGCGCCACCCTCGAGCATTTCGGCCGGCGCTTTCTCAC
18              GCCGCGCCCCGCCGCCACGCTGAGCGCCACCCTCGAGCATTTCGGCCGGCGCTTCCTCAC
                ****************************************************** *****

PAO1            CAGCCTGCTCGATCCCCGCTGCCAGAGCCTCTATCGCCTGGTGGTGGCGGAATCCCCGCG
19              CAGCCTGCTCGATCCCCGCTGCCAGAGCCTCTATCGCCTGGTGGTGGCGGAATCCCCGCG
18              CAGCCTGCTCGATCCCCGCTGCCAGAGCCTCTATCGCCTGGTGGTGGCGGAATCTCCGCG
                ****************************************************** *****

PAO1            GTTTCCGGCGATCGGCAAGTCCTTCTACGAGCAGGGGCCGCAGCAGAGCTATCTGCTGCT
19              GTTTCCGGCGATCGGCAAGTCCTTCTACGAGCAGGGGCCGCAGCAGAGCTATCTGCTGCT
18              GTTTCCGGCGATCGGCAAGTCCTTCTACGAGCAGGGGCCGCAGCAGAGCTACCTGCTGCT
                *************************************************** ********

PAO1            CAGCGAGCGACTGGCCGCGGTCGCTCCTCACATGGACGAGGAAACGCTCTACGCGGTGGC
19              CAGCGAGCGACTGGCCGCGGTCGCTCCTCACATGGACGAGGAAACGCTCTACGCGGTGGC
18              CAGCGAGCGACTGGTAGCGGTCGCCCCCCACATGGACGAGGAAACGCTCTACGCGGTGGC
                **************  ******** ** ********************************

PAO1            CTGCCAGTTTCTCGAGATGCTCAAGGCCGACCTGTTCCTCAAGGCCCTCAGCGTGGCCGA
19              CTGCCAGTTTCTCGAGATGCTCAAGGCCGACCTGTTCCTCAAGGCCCTCAGCGTGGCCGA
18              CTGCCAGTTTCTCGAGATGCTCAAGGCCGACCTGTTCCTCAAGGCCCTCAGCGTGGCCGA
                ************************************************************

PAO1            CTTCCAGCCGACCATGGCGCTGCTGGAAACCCGCCTCAAGCTGTCGGTGGACATCATCGC
19              CTTCCAGCCGACCATGGCGCTGCTGGAAACCCGCCTCAAGCTGTCGGTGGACATCATCGC
18              CTTCCAGCCGACCATGGCACTGCTGGAAACCCGCCTCAAGCTGTCGGTGGACATCATCGC
```

```
                  ****************** *****************************************

PAO1              CTGCTACCTGGAACACCTGTCGCAGAGCCCCGCGCAGGGCTGA
19                CTGCTACCTGGAACACCTGTCGCAGCGCCCCGCGCAGGGCTGA
18                CTGCTACCTGGAACACCTGTCGCAGCGCCCCGCGCAGGGCTGA
                  ************************* *****************
```

mexR

```
CLUSTAL 2.1 multiple sequence alignment


PAO1              ATGAACTACCCCGTGAATCCCGACCTGATGCCCGCGCTGATGGCGGTCTTCCAGCATGTG
18                ATGAACTACCCCGTGAATCCCGACCTGATGCCCGCGCTGATGGCGGTCTTCCAGCATGTA
1                 ATGAACTACCCCGTGAATCCCGACCTGATGCCCGCGCTGATGGCGGTCTTCCAGCATGTG
                  *********************************************************** 

PAO1              CGGACGCGCATCCAGAGCGAGCTCGATTGCCAGCGACTCGACCTGACCCCGCCCGACGTC
18                CGGACGCGCATCCAGAGCGAGCTCGATTGCCAGCGACTCGACCTGACCCCGCCCGACGTC
1                 CGGACGCGC-----------GCTCGATTGCCAGCGACTCGACCTGACCCCGCCCGACGTC
                  *********           ****************************************

PAO1              CATGTATTGAAGCTTATCGACGAACAACGCGGGCTGAACCTGCAGGACCTGGGACGCCAG
18                CATGTATTGAAGCTTATCGACGAACAACGCGGGCTGAACCTGCAGGACCTGGGACGCCAG
1                 CATGTATTGAAGCTTATCGACGAACAACGCGGGCTGAACCTGCAGGACCTGGGACGCCAG
                  ************************************************************

PAO1              ATGTGCCGCGACAAGGCACTGATCACCCGGAAGATCCGCGAGCTGGAGGGAAGAAACCTG
18                ATGTGCCGCGACAAGGCACTGATCACCCGGAAGATCCGCGAGCTGGAGGGAAGAAACCTG
1                 ATGTGCCGCGACAAGGCACTGATCACCCGGAAGATCCGCGAGCTGGAGGGAAGAAACCTG
                  ************************************************************

PAO1              GTCCGCCGCGAGCGCAACCCCAGCGACCAGCGCAGCTTCCAGCTCTTCCTCACCGACGAG
18                GTCCGCCGCGAGCGCAACCCCAGCG-------CAGCTTCCAGCTCTTCCTCACCGACGAG
1                 GTCCGCCGCGAGCGCAACCCCAGTGACCAGCGCAGCTTCCAGCTCTTCCTCACCGACGAG
                  ***********************       ******************************

PAO1              GGGCTGGCCATCCACCAGCATGCGGAGGCCATCATGTCACGCGTGCATGACGAGTTGTTT
18                GGGCTGGCCATCCACCAGCATGCGGAAGCCATCATGTCACGCGTGCATGACGAGTTGTTT
1                 GGGCTGGCCATCCACCAGCATGCGGAAGCCATCATGTCACGCGTGCATGACGAGTTGTTT
                  ************************** *********************************

PAO1              GCCCCGCTCACCCCGGTGGAACAGGCCACCCTGGTGCATCTCCTCGACCAGTGCCTGGCC
18                GCCCCGCTCACCCCGGAGGAACAAGCCACCCTGGTGCATCTCCTCGACCAATGCCTGGCC
```

```
1               GCCCCGCTCACCCCGGAGGAACAAGCCACCCTGGTGCATCTCCTCGACCAATGCCTGGCC
                **************** ****** ************************** *********

PAO1            GCGCAACCGCTTGAGGATATTTAA
18              GCGCAACCGCTTGAGGATATTTAA
1               GCGCAACCGCTTGAGGATATTTAA
                ************************
```
